# Targeting shared molecular etiologies to accelerate drug development for rare diseases

**DOI:** 10.15252/emmm.202217159

**Published:** 2023-06-27

**Authors:** Galliano Zanello, Macarena Garrido‐Estepa, Ana Crespo, Daniel O'Connor, Rima Nabbout, Christina Waters, Anthony Hall, Maurizio Taglialatela, Chun‐Hung Chan, David A Pearce, Marc Dooms, Philip John Brooks

**Affiliations:** ^1^ Institut National de la Santé et de la Recherche Médicale Paris France; ^2^ Instituto de Salud Carlos III (ISCIII) Madrid Spain; ^3^ Sanofi, Specialty Care Milan Italy; ^4^ Medicines and Healthcare Products Regulatory Agency (MHRA) London UK; ^5^ Department of Pediatric Neurology, Reference Center for Rare Epilepsies, Hôpital Necker‐Enfants Malades, Institut Imagine, INSERM U1163 Université Paris Cité Paris France; ^6^ RARE Science, Inc Encinitas CA USA; ^7^ Healx Ltd. Cambridge UK; ^8^ Department of Neuroscience University of Naples Federico II Naples Italy; ^9^ Sanford Research Sioux Falls SD USA; ^10^ Sanford School of Medicine University of South Dakota Sioux Falls SD USA; ^11^ Hospital Pharmacy University Hospitals Leuven Leuven Belgium; ^12^ National Center for Advancing Translational Sciences National Institutes of Health Bethesda MD USA

**Keywords:** basket clinical trials, IRDiRC, rare diseases, shared molecular etiologies, therapeutic development, Genetics, Gene Therapy & Genetic Disease

## Abstract

Rare diseases affect over 400 million people worldwide and less than 5% of rare diseases have an approved treatment. Fortunately, the number of underlying disease etiologies is far less than the number of diseases, because many rare diseases share a common molecular etiology. Moreover, many of these shared molecular etiologies are therapeutically actionable. Grouping rare disease patients for clinical trials based on the underlying molecular etiology, rather than the traditional, symptom‐based definition of disease, has the potential to greatly increase the number of patients gaining access to clinical trials. Basket clinical trials based on a shared molecular drug target have become common in the field of oncology and have been accepted by regulatory agencies as a basis for drug approvals. Implementation of basket clinical trials in the field of rare diseases is seen by multiple stakeholders—patients, researchers, clinicians, industry, regulators, and funders—as a solution to accelerate the identification of new therapies and address patient's unmet needs.

## Graphical abstract

This Review discusses challenges and opportunities in implementing basket clinical trials to accelerate the identification of new therapies for rare diseases.

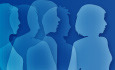

**Glossary**
Basket clinical trial: A basket clinical trial is a type of clinical trial designed to evaluate a single treatment intervention for multiple diseases that share common molecular alteration.
IRDiRC: International Rare Diseases Research Consortium.
Rare diseases: Rare diseases are diseases which affect a small number of people compared to the general population (<200,000 in the United States, <1 in 2,000 people in the European Union, <50,000 or 1 in 2,500 people in Japan) and specific issues are raised in relation to their rarity.
Shared molecular etiologies: This concept refers to molecular alterations that are shared by a number of different diseases.

## Introduction

Rare diseases, defined as affecting <200,000 in the United States or <1 in 2,000 people in the European Union, are uncommon when considered individually. Taken together, the >10,000 rare diseases affect over 400 million people worldwide representing 3.5–5.9% of the world's population (Global Genes: RARE Disease Facts, n.d.; Nguengang Wakap *et al*, 2020). It is therefore accurate to consider that rare diseases are prevalent and represent a real public health issue, with considerable social and economic impact. The majority of conditions have pediatric onset and less than 5% of rare diseases have an approved treatment with orphan drugs. When a diagnosis can be made, many patients are faced with the fact that no treatment exists for their condition. Several legislations starting with the US Orphan Drug Act in 1983 were created to incentivize the development of orphan drugs. Despite the efforts of policy makers, research investigators, patient groups and biopharmaceutical industries, it is estimated that an average of only 50 orphan medicinal products are approved each year for the treatment of rare diseases in the EU and/or USA (RD Metrics – IRDiRC, n.d.). Moreover, many of these new products target diseases already served by approved therapeutic options leaving patients' unmet needs unaddressed (List_of_orphan_drugs_in_europe.pdf, n.d.).

Several challenges limit therapeutic development for rare diseases. The patient populations are typically small, heterogeneous, and geographically dispersed. Diagnosis of rare diseases are typically delayed, limiting collection of valuable medical data, and delaying initiation of appropriate therapeutic measures. Knowledge about the natural history of the disorders is often limited and rare diseases present variable progression across the lifespan of the patients, and, often, across a wide spectrum of disease burden. There is a lack of validated biomarkers, clinical outcomes and endpoints to measure the safety and efficacy of clinical intervention. Identification, inclusion, and retainment of patients in clinical trials represent another difficulty. These hurdles increase the complexity, the duration and the cost of therapeutic development making this process extremely uncertain and with a prominent level of risk for drug developers.

Providentially, the progress in genomics and the implementation of precision‐based medicine opens a path toward the development of new therapies targeting the specific molecular alteration of rare disease patients (Might & Crouse, 2022). Grouping rare disease patients based on the underlying molecular etiology, rather than the traditional, symptom‐based definition of disease, has the potential to identify groups of diseases likely to respond to the same agent, allowing for innovative approaches, which may greatly increase the number of patients gaining access to clinical trials. It also shows the potential to accelerate drug development and to faster address the needs of millions of patients with rare and ultra‐rare diseases (Brooks *et al*, 2014).

Basket clinical trials are designed to evaluate a single treatment intervention for multiple diseases that share common molecular alterations (Woodcock & LaVange, 2017). Ongoing efforts in research and drug development are targeting these shared molecular etiologies. For example, the National Center for Advancing Translational Sciences opened funding opportunities to support basket clinical trials of drugs targeting shared molecular etiologies in more than one rare disease (RFA‐TR‐20‐031: Basket Clinical Trials of Drugs Targeting Shared Molecular Etiologies in Multiple Rare Diseases (UG3/UH3 Clinical Trial Required), n.d.). Importantly, the basic approach of enrolling patients in basket clinical trials based on a molecular marker, rather than clinical signs and symptoms, became more common in the field of oncology, and has been accepted by regulatory agencies, resulting in drug approvals (Park *et al*, 2020).

The International Rare Diseases Research Consortium (IRDiRC) established a Task Force with the objectives to address and document the existing challenges and opportunities in adapting the basket trial approach used in molecularly targeted oncology clinical trials to drugs targeting shared molecular etiologies underlying multiple rare diseases (outside rare cancer indications). This article presents the recommendations of the Task Force and its views regarding future implications for rare disease drug development. Task Force recommendations are broadly disseminated by IRDiRC for consideration by the public and other relevant stakeholders.

## Shared molecular etiology approach in rare diseases: definition, challenges and opportunities

### Definition

Precision medicine is an innovative approach for disease treatment and prevention that takes into account individual molecular alterations in a patient or a sub‐group of patients. If we consider the development of therapies for one rare disease at the time, it becomes evident that the conduct of “precision medicine”—clinical trials in rare disease patients will exacerbate even more the challenges associated with small population studies (e.g., small number of patients, methodological limitations, and requirement for multiple trials to demonstrate efficacy). The master protocol framework offers an opportunity to overcome some of these hurdles and answer more questions in a shorter time. The term master protocol refers to a study design that uses one overarching protocol to guide multiple, simultaneously occurring sub‐studies (Woodcock & LaVange, 2017; Meyer *et al*, 2020). This broad definition includes three types of study design named basket, umbrella, and platform trials. The objective of basket clinical trials is to assess the efficacy of one targeted therapy for multiple diseases. This study design could increase the inclusion in clinical trials of a higher number of rare disease patients sharing a common molecular alteration. The objective of umbrella clinical trials is to assess the efficacy of multiple targeted therapies in the context of one disease. A platform trial is designed to assess multiple therapies in the context of one disease, with the flexibility to drop or add new arms.

**Figure 1:**
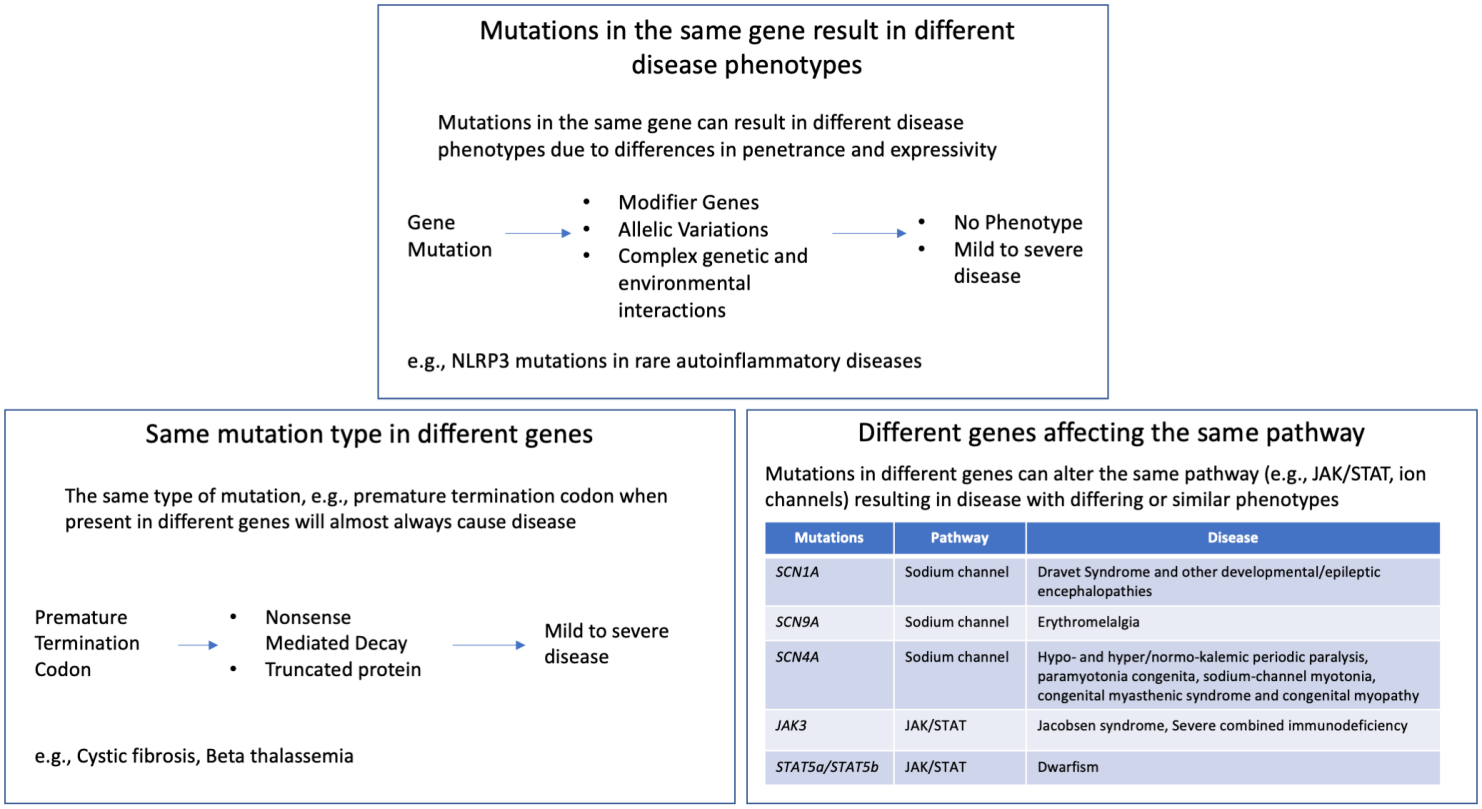
Categorizing rare diseases sharing the same molecular etiology Figure 1 represents categories of rare diseases characterized by overarching types of molecular alterations for which basket clinical trials could be considered.

The aim of the IRDiRC Task Force is to identify common opportunities, challenges and roadblocks to such rare disease basket trials which utilize the same drug for multiple diseases, and propose solutions to overcome them. To clarify the concept of shared molecular etiologies (SaME), the Task Force group identified three categories of rare diseases characterized by overarching types of molecular alterations. Basket trials could be designed to assess a single treatment intervention for multiple rare diseases in each category (see Fig 1).

- The first category defines rare diseases characterized by mutations in the same gene (e.g., NLRP3 mutations are associated with a group of rare hereditary autoinflammatory diseases called cryopyrin‐associated periodic syndromes) (Conforti‐Andreoni *et al*, 2011). Such mutations can result in different disease phenotypes due to differences in penetrance and expressivity. Targeting the defective gene could therefore benefit patients with different diseases.
- The second category defines rare diseases characterized by the same type of mutation (e.g., premature termination codon mutation) on different genes. Premature termination codon mutations are responsible for about 10% of all cases of monogenic diseases (Mort *et al*, 2008). The delivery of suppressor‐tRNA presents a great opportunity to overcome these mutations (Wang *et al*, 2022).
- The third category defines rare diseases characterized by mutations in different genes affecting the same molecular pathway (e.g., mTOR and JAK/STAT) and resulting in diseases with differing or similar phenotypes (Rosner *et al*, 2008; Banerjee *et al*, 2017).

It is important to highlight that these categories are not always independent from each other as different variants in one gene (category 1) can lead to the same altered molecular pathway (category 3). The distinction made by the Task Force between the categories is intended to emphasize on therapeutic development since different categories can lead to very different targeted interventions when considering the technological and biological approaches (e.g., ATMP versus drug).

The Task Force identified a supplementary category to be included in a basket trial approach but differing from the other categories as it does not target a SaME but rather a similar clinical pathophysiology. For example, mutations in different, unrelated genes leading to different diseases can often share similar clinical manifestations such as seizures (Rare and Complex Epilepsies—Find Our More on Epilepsies, n.d.; McTague *et al*, 2016). Although very different from the concept of SaME, inclusion of this additional category in the basket trial approach may also accelerate drug development for a variety of rare diseases and address unmet needs in different patient populations.

### Challenges

Challenges in the conduct of clinical trials in rare diseases are myriad. In the context of the SaME basket clinical trial approach, it will be essential to overcome the limitations associated with small patient populations, particularly when considering ultra‐rare diseases, clinical heterogeneity in rare disease patients affected by the same or different disease, and different outcome measures in the design and the analysis of basket clinical trials. Identification of biomarkers, clinical outcomes and endpoints to measure response to treatment remains an important limitation due to the lack of natural history knowledge in many rare diseases. Importantly, the SaME approach does not obviate the requirement for validated outcome measures. While the outcome measures may be different for each disease, all outcome measures must be suitable for use in a clinical trial, regardless of whether it is a basket trial or a traditional, single disease trial. Another challenge is to evaluate the biologic plausibility of the targeted intervention strategy, define the strength of association of shared molecular entity across different clinical conditions and understand the spectrum of drug pharmacological action to always allow extrapolation from one condition to another. Safety in different populations with different symptoms and existing co‐morbidities is an additional factor to consider before including patients in the study and assessing the efficacy of the targeted therapy. Finally, efforts will be required to reach common agreement of relevant stakeholders including clinical investigators, centers of excellence, patient groups, regulators, and payers (Health Technology Assessment).

### Opportunities

The SaME basket clinical trial approach can provide many opportunities to accelerate drug development for rare disease patients and address their unmet needs. One benefit is to potentially increase the number of patients gaining access to clinical trials and limit the methodological and analytic hurdles associated with studies involving small populations. The SaME approach could also favor a clustering of patients based on their molecular signature and stimulate innovation through the development of precision‐based medicines addressing the needs of patients with different rare diseases. Such efforts are already tackled by the PRECISEADS Consortium allowing the reclassification of individuals affected by systemic autoimmune diseases into four different clusters of molecular groupings (Barturen *et al*, 2018). Several drugs used to treat multiple rare diseases were evaluated for one disease at the time and received marketing authorization at different dates, for example, glycerol phenylbutyrate/six EMA indications; metreleptin/four EMA indications; canakinumab and anakinra/seven EMA indications (EMA, 2018a, 2018b, 2018c, 2018d). The SaME approach could also accelerate the extensive and rapid approval and use of existing drugs that are currently not accessible to patients that would benefit from their action. Additionally, it has the potential to decrease the administrative and operation costs associated with the conduct of multiple clinical trials assessing the efficacy of a therapy for one disease at the time. Finally, although this point was previously listed as a challenge, the SaME approach can foster engagement of multiple stakeholders and stimulate transversal activities in clinical research networks that are structured to facilitate clinical trial readiness and accelerate the development of innovative therapeutic solutions.

## Key considerations for the implementation of the basket trial approach in rare diseases

**Figure 2:**
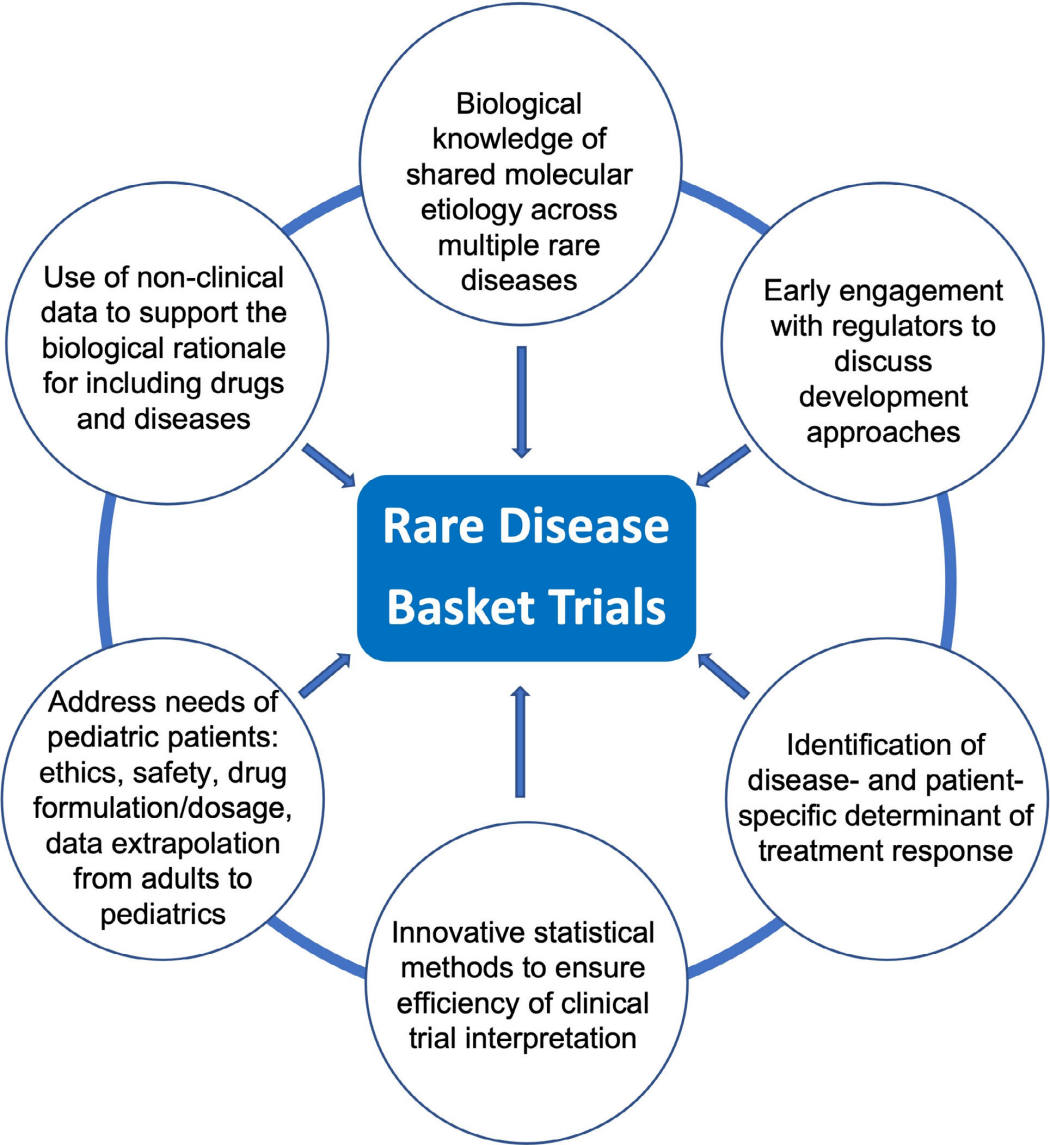
Key considerations for successful design and implementation of basket trials in rare diseases Figure 2 summarizes the key considerations identified by the Task Force for successful design and implementation of basket trials in rare diseases.

Patients enrolled in a SaME basket clinical trial will represent multiple rare diseases characterized by a common molecular alteration. The basket trial approach raises critical issues that need to be carefully considered in order to ensure a qualitative trial design, its authorization by regulatory bodies and efficiency of data interpretation. The Task Force identified the following considerations to frame a basket clinical trial approach in rare diseases. Figure 2 summarizes these considerations for successful design and implementation of basket trials.

- Sufficient understanding of the biology across multiple rare diseases is essential to provide scientific evidence for developing a drug targeting SaME (e.g., molecular etiology and pathophysiology of the rare diseases, gain or loss‐of‐function mutations, validated biomarkers and/or clinical outcomes, drug's mechanism of action). This highlights the importance of a continued collaboration between basic science research groups, clinical research, patients and trial sponsors, as these learnings will contribute to a better perception, interpretation and classification of different rare diseases, and, thus, a more methodic grouping for potential joint assessment.
- Non‐clinical data (*in vitro*, *in vivo*, and computational approaches) may be used to support the biological rationale for a drug's effect across rare diseases sharing the same molecular etiology and provide support for the implementation of basket clinical trials. In addition to the importance of basic research, the latest development of Artificial Intelligence and Machine Learning are promising approaches both for defining groups of rare disease to be tested in the same trial, but also to identify drugs that may have a therapeutic effect in a certain group of diseases. The latter may be an influencing factor for testing drugs originally intended for larger non‐rare populations in rare disease populations with plausible disease‐modifying effects.
- Understanding how the molecular alteration influences the natural history of the disease across the lifespan (i.e., disease progression, age‐related symptoms, and genotype/phenotype correlation) may provide supportive information regarding the prognosis of subjects with a particular molecular alteration as compared to those with the same rare disease who do not harbor the same alteration. Establishing rare disease registries may lead to improved knowledge of these aspects and contribute to establishing prognostic relationships.
- Determining if patient factors and variable disease progression having a large spectrum of genotype–phenotype correlations and/or existing co‐morbidities can result in different response to treatment within and across rare diseases.
- Determining if patient factors in one or more sub‐groups may affect drug effectiveness.
- Under optimal conditions, each sub‐study of a basket clinical trial should include specific objectives, the scientific rationale for inclusion of each population, and a detailed statistical analysis plan. This approach will allow for identification of a partial success, without compromising the whole trial if a mixed outcome (positive and negative) is observed, lessening the risk for trial sponsors. In certain cases, this may not be possible, in which case such sub‐studies would be viewed as exploratory/proof of concept studies, rather than hypothesis testing.
- Early engagement with the regulators to discuss the development approaches including the selection of patients, the scientific rationale to group patients, the ability to extrapolate the data from the pooled analysis, the selection and validation of biomarkers and clinical endpoints for the study design, and the statistical methodologies to assess drug safety and efficacy. This will certainly lead to protocol methodology improvements, particularly considered the requirement for innovative and/or complex designs.
- The selection of biomarkers and clinical endpoints in the basket clinical trial can be specific to each rare disease. However, if the selection is common to the group of diseases, the efficacy results should not be weighted toward a specific rare disease.
- As much as possible, basket clinical trials should address the needs of pediatric rare disease patients sharing the same molecular alteration. Consultation with the regulators regarding pediatric studies should be initiated as early as possible in the drug development (i.e., ethical considerations, safety considerations, drug formulation and dosage, and data extrapolation from adult to pediatric).
- Although basket clinical trials bear the potential to overcome some difficulties associated with small populations, the study design and the methodological approaches should be closely discussed and validated with statistical experts and regulators in an early dialog to ensure successful interpretation of the study and accurate and balanced data analysis.
- Application of innovative statistical methodologies rather than classical methods can improve the efficiency of the clinical trial interpretation. For example, sample size, randomization and early study‐arm termination can be optimized by using Bayesian models and performing mid‐term analyses (Kidwell *et al*, 2022). Evaluation of the intervention effectiveness can incorporate novel approaches such as Multi‐domain responder index which evaluates a broad array of complex and heterogeneous disease manifestations by summing the scores corresponding to clinically significant thresholds of change for each component domain in each individual patient, capturing the mean clinically meaningful change across multiple domains within individuals (Tandon & Kakkis, 2021).

## Examples of basket clinical trials in rare diseases

Table 1 shows some examples of basket clinical trials in rare diseases (outside rare cancers) identified by the Task Force across public databases and that are still ongoing or have been already completed. These examples demonstrate that a basket trial study design can be implemented in rare diseases and offer a basis for the future development of studies in this field.

**Table 1:** Examples of basket clinical trials in rare diseases.

Clinical trial N° (www.clinicaltrials.gov)	Targeted rare diseases	Intervention	Status	Phase	Outcome	Other data
NCT04221451: A multinational, randomized, double‐blind, placebo‐controlled study to assess the efficacy, pharmacodynamics, pharmacokinetics, and safety of venglustat in late‐onset GM2	Primary population: GM2 gangliosidosis (Tay‐Sachs disease or, Sandhoff disease) Secondary population: ultra‐rare diseases within the same and similar glucosylceramide‐based sphingolipid pathway (GM1 gangliosidosis, saposin C deficiency, sialidosis type 1 or galactosialidosis)	Drug: Venglustat (glucosylceramide synthase inhibitor) Drug: Placebo	Active, not recruiting	Phase 3	In Primary population: Percent change to baseline in cerebrospinal fluid (CSF) GM2 biomarker Annualized rate of change in the 9‐hole pegboard test from baseline Assessment of pharmacodynamic response of several biomarkers in plasma and CSF Safety/tolerability: Adverse events	The total duration is up to approximately 223 weeks, with 104‐week primary analysis treatment period, a 104‐week open‐label extension treatment period and a 6‐week post‐treatment safety observation period
NCT02059291: Study of efficacy and safety of canakinumab in patients with hereditary periodic fevers	Colchicine‐resistant familial Mediterranean fever, mevalonate kinase deficiency or, tumor necrosis factor receptor associated periodic syndrome (TRAPS)	Drug: Canakinumab (interleukin‐1β blocker) Drug: Placebo	Completed	Phase 3	Primary outcome measures: Percentage of participants with resolution of initial flare and absence of new flares up to the end of the randomized treatment epoch (16 weeks)	This study includes pediatric population: Inclusion criteria: Male and female patients at least 2 years of age at the time of the screening visit. Male and female patients >28 days but <2 years eligible for open label treatment only
NCT03759639: N‐Acetyl‐L‐Leucine for Niemann‐Pick disease; NCT03759665: N‐Acetyl‐L‐Leucine for GM2 gangliosdisosis (Tay‐Sachs and Sandhoff disease) and; NCT03759678: N‐Acetyl‐L‐Leucine for Ataxia‐Telangiectasia (A‐T)	Niemann‐Pick type C; GM2 gangliosidosis or, Tay‐Sachs disease; Sandhoff disease, ataxia telangiectasia or, Louis Bar syndrome	Drug: N‐Acetyl‐L‐Leucine (multi‐modal mechanism of action, including altered glucose and antioxidant metabolism, reduced lysosomal storage, reduction of neuroinflammation in the cerebellum, leading to the attenuation of cell death)	Active, only NCT03759678 is still recruiting.	Phase 2	Primary outcome measures: Clinical Impression of Change in Severity (CI‐CS) [Time Frame: CI‐CS comparing Baseline (Day 1)] with IB1001 verses the end of 6‐weeks treatment with IB1001 (Approximately Day 42) MINUS the CI‐CS comparing the end of 6‐weeks treatment with IB1001 (Approximately Day 42) versus the end of 6‐weeks post‐treatment washout	These studies include pediatric population: Inclusion criteria: Male or female aged ≥6 years in Europe OR ≥18 years in the United States with a confirmed diagnosis of NPC or GM2 Gangliosidosis, or ≥6 years with a confirmed diagnosis of A‐T at the time of signing informed consent.
NCT02502903: Safety, tolerability and activity of BIVV009 in healthy volunteers and patients with complement mediated disorders	Bullous pemphigoid (BP), cold agglutinin disease (CAD), warm autoimmune hemolytic anemia (WAIHA) or, end‐stage renal disease (ESRD).	Drug: Sutimlimab (C1s blocker) Other: Placebo	Completed	Phase 1	Primary outcome measures: Drug‐related adverse event profile of BIVV009 [Time Frame: 6 weeks]	Apart from safety, the secondary endpoints of the study are to evaluate the pharmacokinetic profile of BIVV009; the classical pathway Complement System activity; the Complement System‐Related biomarkers; the Coagulation System‐Related biomarkers and; the Disease‐Related Biomarkers.
NCT04988087: A study to evaluate the safety, tolerability and efficacy of MHV370 in participants with Sjogren's syndrome (SjS) or mixed connective tissue disease (MCTD)	Sjogren's syndrome (SjS) or, mixed connective tissue disease	Drug: MHV370 (unknown mechanism of action) Drug: Placebo	Recruiting	Phase 2	Primary outcome measures: SjS participants: Change from baseline in Eular Sjögren's Disease Activity Index (ESSDAI) after 24 weeks of treatment [Time Frame: baseline, Week 24] MCTD participants: Change from baseline in physician's global assessment scale (PhGA) after 24 weeks of treatment [Time Frame: baseline, Week 24]	Estimated study completion date is January 31, 2024
NCT04966741: A study to evaluate the efficacy, safety and tolerability of setmelanotide over 1 year of treatment, in pediatric patients aged 2 to <6 years with obesity due to either biallelic variants of the POMC, PCSK1 or LEPR genes or Bardet‐Biedl Syndrome (BBS)	Bardet‐Biedl syndrome, POMC deficiency obesity, PCSK1 deficiency obesity or, LEPR deficiency obesity	Drug: Setmelanotide (MC4R agonist)	Active, not recruiting	Phase 3	Primary outcome measures: Proportion of patients demonstrating >0.2 decrease from baseline in body weight [Time Frame: Baseline to Week 52] Mean percent change in BMI [Time Frame: Baseline to Week 52]	This study includes pediatric population

As mentioned above in the text, some rare disease specific features pose a challenge for the implementation of basket trials (small patient population size, clinical heterogeneity of phenotypes, and selection of clinical outcome measures). For that reason, it was very interesting to understand how the studies already identified have managed to resolve those potential challenges.

As observed in Table 1, five of the six studies—NCT04221451, NCT02059291, NCT02502903, NCT04988087, and NCT04966741—(Novartis Pharmaceuticals, 2018, 2023; Bioverativ, a Sanofi company, 2022; Genzyme, a Sanofi Company, 2022; Rhythm Pharmaceuticals, Inc, 2022) were planned since the beginning as a unique basket clinical trial, while one of them (N‐Acetyl‐L‐Leucine efficacy in different rare diseases) was developed as a three‐step study—NCT03759639, NCT03759665, and NCT03759678—(IntraBio Inc, 2022a, 2022b, 2022c) sharing the same outcome measure. It is important to mention that despite the rare disease limitation in terms of number of patients recruited, most of the studies were designed as placebo‐controlled studies.

Another concern raised by the joint evaluation of multiple rare diseases is the clinical heterogeneity between them and how to evaluate the results. In that line, five of the studies evaluated efficacy in addition of safety—NCT04221451, NCT02059291, NCT04988087, [NCT03759639, NCT03759665, NCT03759678], and NCT04966741—while the study NCT02502903, a phase 1 study, included as primary endpoints only the safety/tolerability of the drug. Most of the studies identified used the same primary outcome for all the diseases included, but study NCT04988087 reflects the capacity of basket trials to adapt to the measurement of different outcomes for the different diseases.

Finally, although the use of master protocols in pediatric drug development has been limited, it is important to note that some of the presented studies include pediatric population—NCT02059291, [NCT03759639, NCT03759665, NCT03759678], and NCT04966741—indicating that joint efforts between regulators, clinicians, patient groups, and industry can support the implementation of basket clinical trials designed to address the needs of pediatric rare disease patients sharing the same molecular alteration (Nelson *et al*, 2022).

All these examples show the efforts made by multiple stakeholders and health authorities to implement basket clinical trials in rare diseases and address patients' unmet needs.

## Future implications

The Task Force described the challenges and opportunities in adapting the basket trial approach to rare diseases and presented its recommendations for a more efficient implementation of this approach. The group also identified that such implementation would have future implications leading to innovation in main areas of the therapeutic development chain including: R&D, Regulatory Science, Health Economics, the pharmaceutical industry, and rare disease patients.

### Implications in R&D

The SaME basket clinical trial approach represents a real opportunity to foster the development of new drugs for rare disease patients and address the Goal 2 of IRDiRC: 1000 new therapies for rare diseases will be approved, the majority of which will focus on diseases without approved options (Vision & Goals – IRDiRC, n.d.). Pairing of drugs targeting key pathways and rare diseases characterized by similar molecular alterations affecting these pathways is very likely to accelerate therapeutic development through the repurposing of drugs or *de novo* development. Genetic network analysis and discovery of new gene pathways open another avenue for the implementation of the SaME approach in the rare disease field.

The SaME approach offers an opportunity for increased collaboration between multiple stakeholders, namely between basic research, clinical research, trial sponsors, regulatory bodies, payers, and patient groups. Particularly for patient groups, this approach will grant an increased engagement in research and subsequent steps of development. Classifying rare diseases based on their molecular alteration or signature can unite disease specific patient groups that would otherwise continue isolated, and make them active contributors to the identification and the inclusion of patients in basket trials, the design of such studies and to better connect clinical research teams across the world.

### Implications in regulatory science

Implementation of the SaME approach in rare disease will encourage regulators to better define and clarify the scientific context for regulatory decision making. For example, the definition of a rare disease by regulatory authorities may be an important consideration for sponsors, for example, inclusion of premature stop codon diseases into one group to increase inclusion of patients into clinical trials could lead to a bigger group that may not be considered as a rare condition from a regulatory perspective. This scenario could potentially impact regulatory implication on orphan drug designation, prevalence criteria and incentives.

The clinical heterogeneity of rare diseases, the different progression, the lack of clear endpoints and the requirements around the strength of biological plausibility for data extrapolation between conditions in the SaME approach are additional factors that would make regulatory assessment and approval difficult. The requirements and the strategy for data collection and assessment will have to be clearly defined by regulators to facilitate study design, patient enrollment and/or engagement in phase 3 clinical trials. Furthermore, it may become pertinent to consider the possibility of conditional drug approvals, granting sponsors access to the market, with pre‐defined requirements for more in‐depth data collection based on the finding of basket trials. In this context, the use of real‐world data could help expedite drug approval for rare and ultra‐rare diseases with no approved therapeutic options.

### Implications in health economics

Multi‐indication pricing is a pharmaco‐economic tool to determine one price for a pharmaceutical product with multiple indications, each with a different prevalence/incidence and so‐called “value‐based” price (Persson & Norlin, 2018). In the European Union, there are examples when an orphan medicinal product received a new indication, the price remained the same (bosentan, carglumic acid, imatinib, nintedanib, and sorafenib) except when it was marketed with a different brand name (everolimus as Afinitor/Votubia). In the event of an orphan medicinal product being authorized after successful basket trials, the same relative price may be determined for all the different indications based on the mean “value‐based price.” Furthermore, in line with recent examples from other areas of innovative pricing solutions, alternatives could be sought for pricing “basket trial derived drugs,” such as a pay for performance approach, or progressive with milestone achievements (e.g., increasing levels with conditional approval, delivery of required data at defined period intervals, and full approval).

### Implications for pharmaceutical industry

The proposed basket trial approach sets a roadmap for improved success, while limiting the risk for industry in the development of medicines for very small patient populations. Combining the expertise from all stakeholders (industry, basic research, academia, and patient groups) has the potential to transform the development of medicines, maximizing innovative solutions and leveraging on complementary sources of knowledge. This effort will ultimately address the unmet needs of patients with rare diseases and improve healthcare practice.

### Implications for rare disease patients

Developing treatments for each of the >10,000 rare diseases, especially for diseases that impact very few patients, is not feasible using the current “one disease at a time” approach. Under that system, there is a real possibility that effective treatments may exist for treating subsets of patients with very low prevalence diseases, but those patients will never get access to that treatment simply because no one will open a clinical trial in such a rare disease. In principle, the SaME approach has the potential to allow such patients to participate in a clinical trial of a drug that might benefit them, which is a clear benefit to such patients.

## Broader considerations

The concept of lumping versus splitting has a long history in the rare disease field (McKusick, 1969), continuing up to the present (Biesecker *et al*, 2021). The SaME basket trial approach as discussed here is clearly an example of lumping, but based upon drug targets for molecular etiologies that are shared across multiple diseases. Such drug targets could be based on traditional methods, and/or novel, “omics” based datasets. The basket trial approach described herein is not based on any particular taxonomy or nosology of rare diseases, nor was it our intention to create a new one. Instead, our goal is very practical and translational; the use of basket trials to facilitate rare disease drug development and increase the access of rare disease patients to clinical trials of drugs that might benefit them.

The primary focus of the IRDiRC Task and literature review was on shared molecular etiologies targeted by small molecule drugs. However, the same basic concept of grouping by therapeutic target could be applied to gene‐targeted therapies. For example, mutations that result in cryptic splice sites could be considered as a shared molecular etiology for antisense oligonucleotides (Kim *et al*, 2019), and transition mutations could be considered as a shared molecular etiology for base editors (Rallapalli & Komor, 2023). The clinical development of prime editing and newer developing technologies (Chen & Liu, 2023) could potentially expand this concept even further. However, one crucial difference is that such gene‐targeted therapies are also sequence specific, which adds a substantial additional layer of practical and regulatory complexity to the basket approach compared to a single drug.

## Conclusions

Grouping rare disease patients based on the underlying molecular etiology or pathways can greatly increase the number of patients gaining access to clinical trials. Basket trials show the potential to accelerate drug development and address the needs of millions of rare disease patients. The use of basket trial design in rare disease drug development is still limited and further efforts and collaboration between clinicians, industry, regulatory bodies, policy makers and patients are needed to overcome the challenges limiting the widespread development of these basket trials in rare diseases.

## Author contributions

**Galliano Zanello**: Project administration, conceptualization, visualization, writing – original draft. **Macarena Garrido‐Estepa**: Conceptualization, visualization, writing – review and editing. **Ana Crespo**: Conceptualization, visualization, writing – review and editing. **Daniel O’Connor**: Conceptualization, writing – review and editing. **Rima Nabbout**: Conceptualization, writing – review and editing. **Christina Waters**: Conceptualization. **Anthony Hall**: Conceptualization, writing – review and editing. **Maurizio Taglialatela**: Conceptualization, writing – review and editing. **Chun‐Hung Chan**: Conceptualization, visualization, writing – review and editing. **David A Pearce**: Project administration, conceptualization, visualization, writing – review and editing. **Marc Dooms**: Project administration, conceptualization, writing – review and editing. **Philip John Brooks**: Project administration, conceptualization, writing – review and editing.

## Disclosure and competing interests statement

The authors declare that they have no conflict of interest.

**Pending issues**

- Knowledge about the biology and the natural history of many rare diseases is still lacking. Continuous research efforts are needed to increase the knowledge required to identify clinical outcome measures necessary to clinical trials, including the SaME basket trial approach.
- Regulatory guidelines and frameworks have been developed by regulatory agencies for tissue agnostic drug development in oncology. Same efforts should be carried to facilitate the development of drugs targeting shared molecular etiologies in rare diseases.
- Relevant stakeholders including clinical investigators, centers of excellence, patient groups, regulators, and payers should join efforts to increase the number of SaME basket trials in rare diseases and ensure that approved medicinal products are accessible to patients.
